# Effects of IL‐32 polymorphisms and IL‐32 levels on the susceptibility and severity of coronary artery disease

**DOI:** 10.1002/jcla.24114

**Published:** 2021-11-19

**Authors:** Susu Jin, Xiujing Liu, Yingying Wang, Jian Yu, Minghua Jiang

**Affiliations:** ^1^ Department of Clinical Laboratory The Second Affiliated Hospital and Yuying Children’s Hospital of Wenzhou Medical University Wenzhou China

**Keywords:** coronary artery disease, interleukin‐32, single nucleotide polymorphism

## Abstract

**Background:**

Interleukin‐32 (IL‐32) has long been proposed as a biomarker for coronary artery disease (CAD). We aimed to evaluate the association between IL‐32 levels and coronary stenosis severity, *IL*‐*32* polymorphisms rs28372698 and rs4786370, and CAD susceptibility.

**Methods:**

A total of 362 patients with definite or suspected CAD that underwent angiography were recruited (CAD group, n = 175; nonobstructive CAD group, n = 56; control group, n = 131). The severity of coronary stenosis was assessed using the Gensini score and the number of diseased vessels. IL‐32 levels were determined using enzyme‐linked immunosorbent assay. Gene polymorphisms were genotyped using PCR and sequencing techniques.

**Results:**

IL‐32 levels were significantly different at different levels of coronary artery stenosis *(p* < 0.05), and logIL‐32 was positively correlated with the Gensini score (*r* = 0.357, *p* < 0.01). Multivariate logistic regression analysis revealed that IL‐32 was independently associated with CAD (OR = 6.526, 95% CI: 3.344–12.739, *p* < 0.01). The receiver operating characteristic analysis revealed the area under the curve for discriminating the CAD and Gensini score were 0.605 and 0.613, respectively. Furthermore, IL‐32 levels were significantly higher before percutaneous coronary intervention (PCI) than at 7 days post‐PCI (*p* = 0.012). The homozygous TT genotype and T allele of rs28372698 were found to be associated with increased risk of CAD, while TT homozygosity and the T allele of rs4786370 with reduced risk of CAD (*p* < 0.05). However, both SNPs had no obvious effect on IL‐32 levels or coronary stenosis severity in patients with CAD.

**Conclusion:**

To the best of our knowledge, our study is the first to show that rs28372698 and rs4786370 are associated with CAD susceptibility in Chinese Han population. We also suggest that plasma IL‐32 levels may be indicative of coronary artery stenosis and the efficacy of PCI and provide guidance for risk stratification and disease management.

## Graphical abstract

Effects of gene polymorphisms and levels of interleukin‐32 on the susceptibility and severity of coronary artery disease.

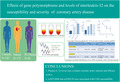

## 1 INTRODUCTION

Coronary artery disease (CAD) is a leading cause of disability and mortality globally.^1^, ^2^ In 2016, 17.9 million people died of cardiovascular disease, accounting for 31% of all deaths worldwide. CAD results from the development of atherosclerosis, and its pathology mainly involves the activation of inflammatory reactions and the coagulation system.^3^ Interleukin‐32 (IL‐32) is a pleiotropic cytokine involved in a number of biological functions, including pro‐ and anti‐inflammatory stimulation, cell differentiation, and apoptosis.^4^ Previous studies have shown that IL‐32 may be involved in the inflammatory cascade that leads to atherosclerosis and contributes to plaque instability by promoting the synthesis of matrix metalloproteinases.^5^ In addition, it has also been described as an activator of p38 mitogen‐activated protein kinase and nuclear factor kappa‐B (NF‐ᴷB) and inducer of several cytokines involved in the process of cardiovascular disease.^6^ IL‐32 may be a new predictor of adverse cardiac events in patients with heart failure after myocardial infarction by upregulating the expression of matrix metalloproteinase‐9 (MMP‐9), procollagen type I (PI), procollagen type III (PIII), and transforming growth factor‐beta (TGFβ) in myocardial fibroblasts.^7^

*IL*‐*32* is located on human chromosome 16 p13.3 and contains single nucleotide polymorphisms (SNPs). rs28372698 and rs4786370 polymorphisms are located in the *IL*‐*32* promoter and have been associated with a variety of diseases, including systemic lupus erythematosus, leishmania infection, and cancer.^8^, ^9^, ^10^ Recently, the AA genotype of rs28372698 polymorphism has been associated with increased cardiovascular mortality.^11^ rs4786370 has a functional effect on the lipid profile of rheumatoid arthritis patients, suggesting a possible protective role against cardiovascular disease (CVD).^12^ Thus, previous reports indicate that polymorphisms in *IL‑32* are important in CAD.

*IL*‐*32* and its SNPs are closely related to the occurrence and progression of cardiovascular diseases. However, studies investigating the relationship between IL‐32 levels and coronary stenosis are rare in patients with CAD, and the role of rs28372698 and rs4786370 in CAD susceptibility has not yet been reported. Therefore, in this study, we analyzed the relationship between plasma IL‐32 levels and coronary atherosclerotic burden, including the number of diseased coronary vessels and clinical risk scores, including the Gensini score, in CAD patients. We also assessed the association between IL‐32 levels and percutaneous coronary intervention (PCI). We hypothesized that SNPs in the *IL*‐*32* promoter would be associated with disease susceptibility in patients with CAD in Chinese Han population.

## 2 MATERIAL AND METHODS

### 2.1 Study subjects

From April 2019 to February 2021, a total of 362 patients who underwent coronary angiography were enrolled in the Second Affiliated Hospital and Yuying Children's Hospital of Wenzhou Medical University. All enrolled subjects were ethnic Han Chinese. CAD was defined as the previous or current detection of at least 50% stenosis of the vessel lumen diameter in one of the main coronary arteries (the right coronary artery, left circumflex, or left anterior descending arteries) by coronary angiography, a previous or current PCI, or a previous or current coronary artery bypass surgery.^13^ Patients with luminal stenosis (1–49% stenosis) were defined as nonobstructive CAD (NO‐CAD).^14^ The subjects with normal coronary arteries were considered as the control group. Based on the clinical symptoms and the results of the coronary arteriogram and electrocardiogram, the CAD patients (n = 175) were divided into stable angina pectoris (SAP, n = 30), unstable angina pectoris (UAP, n = 57), and acute myocardial infarction (AMI, n = 88) groups. Patients with heart failure, valvular heart disease, tumor, liver disease, kidney disease, or immune disease were excluded from the study. Coronary angiography was performed by two experienced interventional cardiologists. In addition, 90 CAD patients who underwent PCI were enrolled. This study was approved by the ethics committee of the Second Affiliated Hospital and Yuling Children's Hospital of Wenzhou Medical University according to the Declaration of Helsinki, and written informed consent was obtained from all participants.

### 2.2 Clinical data collection and blood sampling

Clinical data, including drinking and smoking history, and medical history of hypertension, hyperlipidemia, and diabetes, were collected by physicians and nurses. Body mass index (BMI) was calculated as weight in kilograms divided by height in meters squared. Hypertension was defined as systolic blood pressure (SBP) and/or diastolic blood pressure (DBP) ≥140 mmHg/90 mmHg on at least three occasions on different days or under treatment with antihypertensive drugs.^15^ Diabetes was defined as randomized blood glucose ≥11.1 mmol/L, fasting blood glucose ≥7.0 mmol/L, blood glucose ≥11.1 mmol/L at 2 h after oral glucose tolerance test (OGTT), or treatment with hypoglycemic agents and insulin.^16^ Dyslipidemia was defined as total cholesterol (TC) >240 mg/dl or serum triglycerides (TG) >150 mg/dl, or high‐density lipoprotein cholesterol (HDL‐C) <40 mg/dl, or low‐density lipoprotein cholesterol (LDL‐C) ≥160 mg/dl, or diagnosis/treatment of dyslipidemia.^17^ At admission, we measured fasting glucose, HDL, TG, TC, low‐density lipoprotein (LDL), lipoprotein(a) [LP (a)], hypersensitive C‐reactive protein (hsCRP), cardiac troponin I (cTnI), and brain natriuretic peptide (BNP) (Siemens, Germany). In addition, the left ventricular ejection fraction (LVEF) was determined using a Philips I E33 ultrasound diagnostic instrument (Philips, the Netherlands). Whole blood samples were collected in EDTA anticoagulant tubes (BD, the USA) the day after admission and on the 1st and 7th day after PCI. Part of the whole blood was directly frozen at −70℃ until DNA extraction. The rest of the sample was centrifuged at 400×*g* for 10 min at room temperature, and the separated plasma was stored at −70℃ until analysis.

### 2.3 Assessment of coronary severity

Coronary severity was assessed using the Gensini score and the number of diseased coronary vessels. The Gensini score was computed by assigning a severity score to each site of coronary stenosis according to the degree of luminal narrowing. These points were multiplied by a factor according to the site of the lesion.^18^ The Gensini score was then categorized into the control group (Gensini score = 0), the mild stenosis group (0 < Gensini score ≤40), and the severe stenosis group (Gensini score >40), as described in previous studies.^19^ Diseased coronary vessels were categorized as single‐, double‐, and triple‐vessel stenosis based on the number of stenotic vessels among the left anterior descending artery, left circumflex artery, and right coronary artery.

### 2.4 Measurement of plasma IL‐32 levels

Plasma levels of IL‐32 were determined using enzyme‐linked colorimetric assay kits (Cat. No. DY3040, R&D Systems, USA) with inter‐assay coefficients of variability (CV) <8% and intra‐assay CV <10%. Absorbance was measured at 470 nm and 540 nm as the measurement and reference wavelengths, respectively, using a microplate reader (Tecan, Switzerland).

### 2.5 Genotyping of two genetic variants of *IL*‐*32*

Genomic DNA was isolated from whole blood using the QIAamp^®^ DNA Blood Kit (Catalogue No. 51106, Qiagen, Germany), according to the manufacturer's instructions. PCR and sequencing techniques were used for genotyping rs28372698 and rs4786370. Primers for PCR were designed using the Primer Premier Software version 5.0 (Premier, Canada). The primer pair F: 5′‐CCCCAAGATTGCTGAGACCA‐3 and R: 5′‐AGGAACTGCCGGACCTAAGAG‐3′ was used to amplify *IL*‐*32* polymorphism rs28372698, and F: 5′‐TGTCTTCAGATGGTGGCCTTTG‐3′ and R: 5′‐ACCTGCCTGCCACTTAGGAG‐3′ to amplify rs4786370. Amplification was performed in a final volume of 50 µl containing 400 ng of genomic DNA, 4 µl of each primer, 0.25 µl of TaKaRa Ex Taq, 5 µl of 10 × Ex Taq Buffer, and 4 µl of dNTP mixture (Catalogue No. RR001A, Takara, Japan), and RNase‐free water was added to bring the volume to 50 µl. The PCR conditions were as follows: 30 cycles of denaturation at 92℃ for 20 s, annealing at 58.8℃ for 30 s, and extension at 72℃ for 60 s, and final holding at 10℃. PCR was conducted in an Eppendorf Mastercycler Gradient (Eppendorf, Germany). The PCR products were then sequenced by Sanger sequencing. (Sangon Biotech Co., Ltd., China).

### 2.6 Statistical analyses

Statistical analyses were performed using the SPSS Statistics software version 22.0 (IBM Corp, USA). Normality was tested using the Kolmogorov‐Smirnov test. Student's *t*‐test was used to compare two independent groups, one‐way ANOVA was used to compare multiple groups that were normally distributed, and the Mann‐Whitney U test was performed to compare two non‐normally distributed groups. The Kruskal‐Wallis test was used to test the differences between different groups. The Hardy‐Weinberg equilibrium was calculated using the chi‐squared test. Spearman analysis was used for correlation analysis. Univariate and multivariate logistic regression analyses were performed to evaluate the risk of CAD. The best cutoff values for plasma IL‐32 were determined by receiver operating characteristic (ROC) analysis and area under the curve (AUC) calculations. Statistical significance was set at *p* < 0.05.

## 3 RESULTS

### 3.1 Clinical characteristics of the participants

Baseline characteristics and sample values are summarized in Table 1.TG, cTnI, Glu, SBP, Gensini score, IL‐32, and the history of hypertension smoking and diabetes were higher, while HDL‐C was lower in the CAD patients than in controls (all *p* < 0.05). Nevertheless, the levels of TG and cTnI in the CAD patients were higher than those in the NO‐CAD group (*p* < 0.05). No significant differences in other clinical characteristics, namely, age, sex, BMI, drinking, dyslipidemia, DBP, TC, LDL‐C, LP (a), hs‐CRP, BNP, and LVEF were found between the three groups (*p* > 0.05; Table 1).

**TABLE 1:** Clinical and laboratory characteristics of the patients

Variables	Controls (*n* = 131)	NO‐CAD (*n* = 56)	CAD (*n* = 175)	*p*‐value
Age (year)	65.9 ± 13.28	64.76 ± 11.14	64.09 ± 11.95	0.519
Sex [male (n, %)]	101 (77.09%)	40 (71.43%)	131 (74.86%)	0.708
BMI (kg/m^2^)	24.96 ± 2.90	25.14 ± 3.18	25.02 ± 3.20	0.704
Smoking [n, (%)]	46 (35.11%)	26 (46.43%)	86 (49.14%)^a^	0.045
Drinking [n, (%)]	25 (19.08%)	10 (17.85%)	42 (24.00%)	0.463
Diabetes [n, (%)]	24 (18.32%)	16 (28.57%)	55 (31.43%)^a^	0.033
Dyslipidemia [n, (%)]	55 (41.98%)	29 (51.78%)	61 (34.90%)	0.068
Hypertension [n, (%)]	80 (61.16%)	42 (75.00%)	132 (75.43%)^a^	0.017
SBP (mmHg)	133.40 ± 18.14	135.60 ± 18.61	140.63 ± 21.66^a^	0.002
DBP (mmHg)	77.58 ± 12.12	78.66 ± 11.75	78.67 ± 12.35	0.645
Triglyceride (mg/dl)	103.54 (72.57–151.33)	93.81 (61.95–140.71)	129.21 (92.92–203.55)^a^, ^b^	<0.001
Total cholesterol (mg/dl)	144.73 (119.58–178.79)	158.67 (126.16–192.33)	162.15 (139.70–196.98)	0.055
HDL‐C (mg/dl)	43.45 (34.61–54.22)	31.15 (41.53–52.30)	36.53 (30.76–43.76)^a^	0.001
LDL‐C (mg/dl)	102.59 (80.22–126.89)	98.73 (74.82–116.86)	92.56 (65.18–129.98)	0.271
LP (a) (mg/L)	139.1 (91.52–242.90)	139.4 (82.37–271.95)	174 (96.92–339.72)	0.169
hsCRP (mg/L)	1.53 (0.96–2.82)	1.53 (0.87–2.81)	1.72 (0.80–7.87)	0.780
BNP (pg/L)	124 (35–485.75)	54 (29–443)	147 (42–462)	0.471
cTnI (ng/mL)	0.012 (0.01–0.015)	0.014 (0.012–0.018)^a^	0.155 (0.012–0.570)^a^, ^b^	<0.001
Glu (mmol/L)	5.17 (4.84–5.76)	5.30 (4.81–6.71)	5.77 (4.91–7.37)^a^	0.005
LVEF (%)	64 (60–67)	64 (59.50–66)	64 (58.7–67)	0.772
Gensini score	0 (0–0)	8 (3–15.75)	35 (18–66)	<0.001
IL−32 (pg/ml)	118.53 (32.75–489.90)	262.86 (35.91–723)	324.25 (171.44–497.25)^a^	0.002

Note

Values are presented as numbers (%) or mean ± SD or median (interquartile range).

Abbreviations: BMI, body mass index; BNP, brain natriuretic peptide; CAD, coronary artery disease; cTnI, cardiac troponin I; DBP, diastolic blood pressure; Glu, glucose; HDL‐C, high‐density lipoprotein cholesterol; hsCRP, high‐sensitivity C‐reactive protein; IL‐32, Interleukin‐32;LDL‐C, low‐density lipoprotein cholesterol; LP (a), lipoprotein (a); LVEF, left ventricular ejection fraction; SPB, systolic blood pressure.

^a^ *p* < 0.05 vs. control group.

^b^ *p* < 0.05 vs. NO‐CAD group.

### 3.2 Plasma IL‐32 levels and coronary severity

**FIGURE 1:**
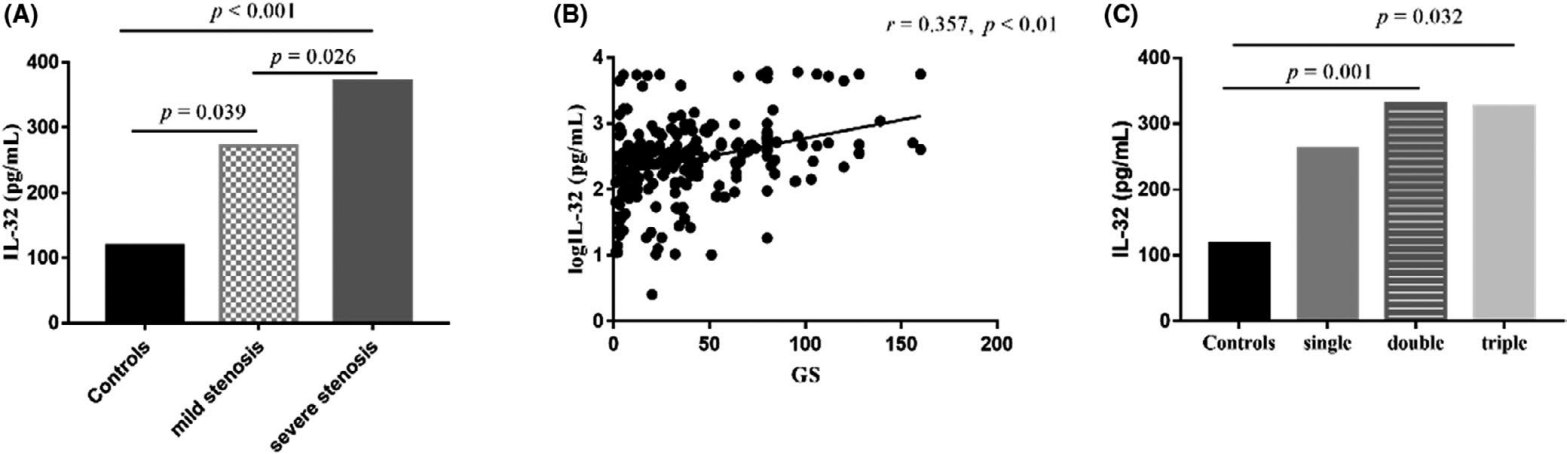
Association between plasma IL‐32 levels and coronary severity. (A) Plasma IL‐32 levels in the control group (Gensini score = 0), the mild stenosis group (0 < Gensini score ≤ 40), and the severe stenosis group (Gensini score > 40). (B) Correlation between logIL‐32 levels and the Gensini score. (C) Comparison of IL‐32 levels with the number of vessels with coronary involvement

In this study, the severity of coronary artery stenosis was assessed based on the number of diseased coronary vessels and the Gensini score. The difference in plasma IL‐32 levels between the three groups was compared, and IL‐32 levels in the severe stenosis group were significantly higher than those in the mild stenosis and the control group (*p* < 0.05). Spearman's correlation analysis showed that plasma IL‐32 levels were positively correlated with the Gensini score (*r* = 0.357, *p* < 0.01; Figure 1B). In addition, subjects with double‐ and triple‐vessel disease showed higher levels of IL‐32 than the controls (*p* = 0.001 and *p* = 0.032, respectively; Figure 1C).

### 3.3 The diagnostic value of IL‐32 in coronary artery disease and coronary stenosis severity

**FIGURE 2:**
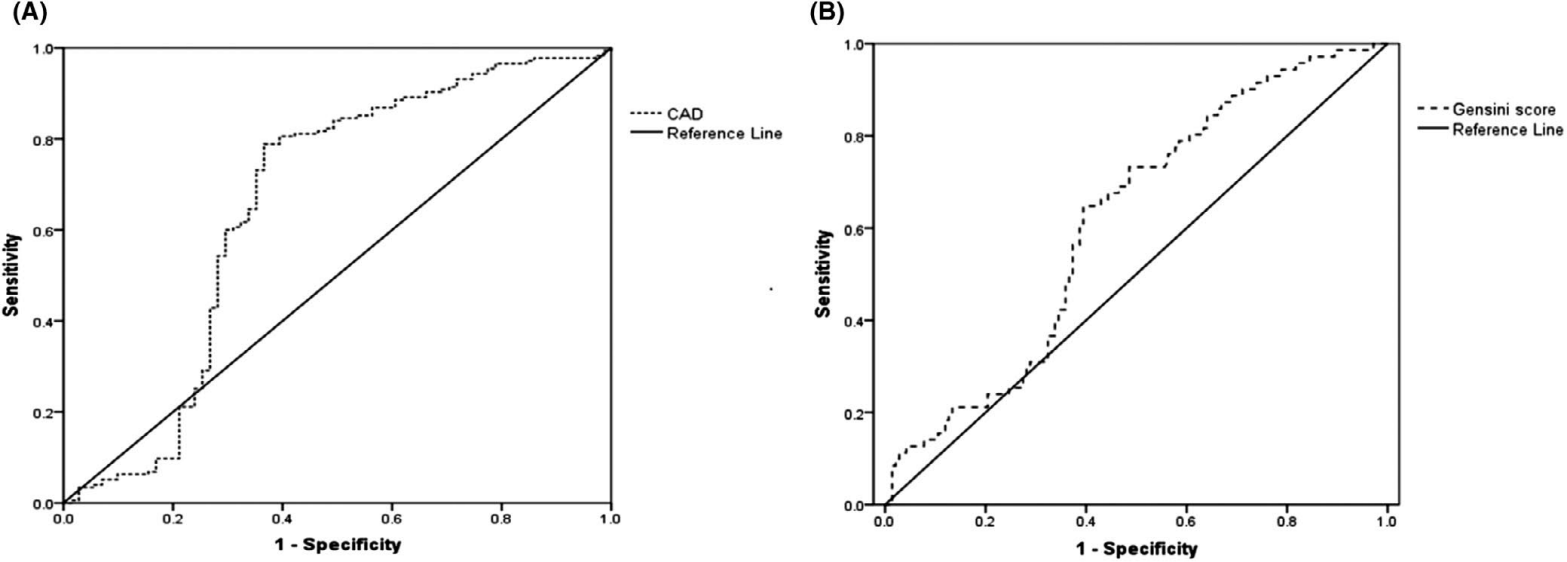
Receiver operating characteristic curve analysis of IL‐32 for the prediction of coronary artery disease (CAD) and Gensini score. (A) Area under the curve (AUC) value of CAD is 0.605 (95% CI: 0.568–0.745, *p* < 0.001). (B) AUC value of Gensini score is 0.613 (95% CI: 0.536–0.690, *p* = 0.007). CAD, Coronary artery disease

Univariate and multivariate logistic regression analyses were conducted to further investigate the risk factors for CAD. As presented in Table 2, logIL‐32 was positively associated with CAD (OR = 11.802, 95% CI: 6.607–21.081, *p* < 0.001). After multivariate adjustment for other variables, the correlation was still significant (OR = 6.526, 95% CI: 3.344–12.739, *p* < 0.001). Additionally, we performed ROC analysis to determine the diagnostic value of plasma IL‐32 levels for CAD. As illustrated in Figure 2A, the highest value of total sensitivity and specificity was set at a cutoff IL‐32 value of 288.32 pg/ml with 58.3% sensitivity and 70.4% specificity. The area under the ROC curve was calculated to be 0.526 (95% CI: 0.568–0.745, *p* < 0.001). We further assessed the use of IL‐32 levels to predict coronary stenosis severity (Gensini scores and diseased coronary vessels). This is illustrated in Figure 2. The area under the curve for the prediction of different grades of significant stenosis was 0.613 (95% CI: 0.536–0.690, *p* = 0.007). However, the AUC for predicting diseased coronary vessels was 0.514 (*p* = 0.672).

**TABLE 2:** Univariate and multivariate logistic regression models for the prediction of coronary artery disease

Variables	Univariate analysis		Multivariate analysis	
	OR (95% CI)	*p*‐value	OR (95% CI)	*p*‐value
Smoking	1.870 (1.307–2.674)	0.001**	0.977 (0.505–1.890)	0.944
Diabetes mellitus	2.391(1.470–3.890)	<0.001**	1.452 (0.572–3.681)	0.432
Hypertension	1.671 (1.264–2.208)	<0.001**	2.440 (1.194–4.990)	0.014*
SBP	1.002(1.001–1.004)	0.004**	0.983 (0.971–0.995)	0.218
Triglyceride	1.246 (1.014–1.532)	0.036*	0.747 (0.535–1.041)	0.085
HDL‐C	0.269 (0.128–0.563)	< 0.001**	0.082 (0.027–0.248)	0.001**
LP (a)	1.001 (1.000–1.002)	0.012*	1.002 (1.000–1.004)	0.011*
Glu	1.206 (1.068–1.362)	0.003**	1.073 (0.898–1.280)	0.438
hs‐CRP	1.121 (1.058–1.188)	<0.001**	1.041 (0.937–1.155)	0.455
logIL−32	11.802 (6.607–21.081)	<0.001**	6.526 (3.344–12.739)	<0.001**

Abbreviations: CAD, coronary artery disease; CI, confidence interval; Glu, glucose; HDL‐C, high‐density lipoprotein cholesterol; hsCRP, high‐sensitivity C‐reactive protein; IL‐32, Interleukin‐32; LP (a), lipoprotein (a); OR, odds ratio; SPB, systolic blood pressure.

* *p* < 0.05

** *p* < 0.01.

### 3.4 Plasma IL‐32 levels in patients with coronary artery disease undergoing percutaneous artery intervention

**FIGURE 3:**
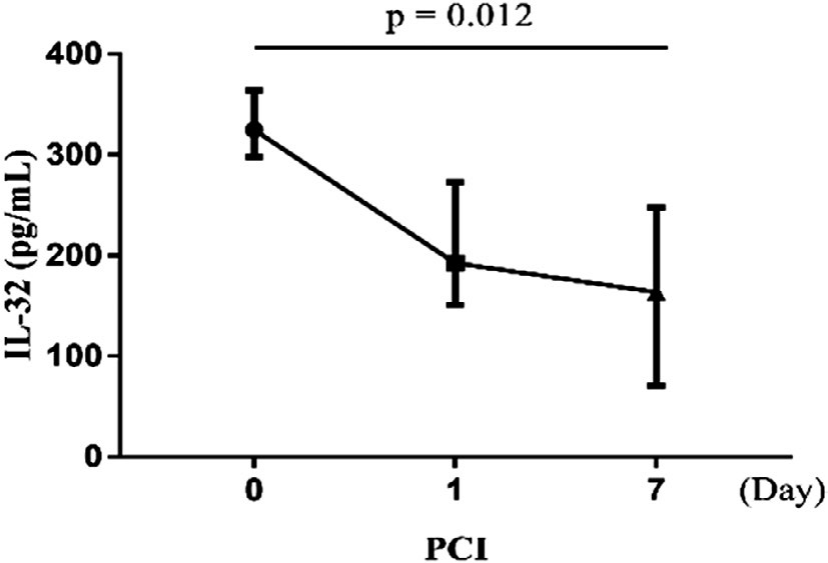
Plasma IL‐32 levels in patients with coronary artery disease undergoing PCI. PCI, percutaneous coronary intervention; IL‐32, Interleukin‐32

Plasma IL‐32 levels in patients that underwent PCI were measured before PCI and at 1 and 7 days post‐PCI. IL‐32 levels at the three timepoints were found to be statistically significant (χ^2^ = 8.312, *p* = 0.016). IL‐32 levels were significantly higher before PCI than at 7 days post‐PCI (*p* = 0.012; Figure 3).

### 3.5 Detection of *IL*‐*32* SNPs and susceptibility to coronary artery disease

rs28372698 and rs4786370 polymorphisms were successfully genotyped in all subjects. The frequencies of both genotypes were in accordance with the Hardy‐Weinberg equilibrium (*p* > 0.05). The allele and genotype frequency of *IL*‐*32* SNPs are presented in Table 3. Significant differences in the genotype and allele distribution of rs28372698 were observed between CAD patients and controls, suggesting that TT homozygosity of rs28372698 increased the risk of CAD by 3.111 (95% CI: 1.206–8.025, *p* = 0.015 in the codominant genetic model) and 3.311 (95% CI: 1.312–8.354, *p* = 0.008 in the recessive genetic model). In addition, the T allele of rs28372698 increased the risk of CAD by 1.459 (95% CI: 1.014–2.099, *p* = 0.041). In contrast, the T allele (OR = 0.636, 95% CI: 0.436–0.930, *p* = 0.019) and TT homozygosity (OR = 0.385, 95% CI = 0.152–0.975, *p* = 0.039 in the codominant) of rs4786370 reduced the risk of CAD. However, there were no significant differences in the genotype frequency of rs4786370 in the dominant, recessive, and overdominant genetic models.

**TABLE 3:** Genotype and allele distribution of *IL*‐*32* SNPs rs28372698 and rs4786370 in patients with coronary artery disease and controls

Model/Allele	Genotype	CAD (*n* = 175)	Controls (*n* = 131)	OR (95% CI)	Test statistic	*p*‐value
rs28372698						
Codominant	AA	90 (51.4%)	75 (57.2%)	Reference	6.588	0.037
	AT	61 (34.9%)	50 (38.2%)	1.017 (0.627–1.649)	0.004	0.947
	TT	24 (13.7%)	6 (4.6%)	3.111 (1.206–8.025)	5.938	0.015
Dominant	AA	90 (51.4%)	75 (57.2%)	Reference		
	AT+TT	85 (48.6%)	56 (42.8%)	1.090 (0.698–1.701)	0.143	0.705
Recessive	AA+AT	151 (86.3%)	125 (95.4%)	Reference		
	TT	24 (13.7%)	6 (4.1%)	3.311 (1.312–8.354)	7.069	0.008
Overdominant	AA+TT	114 (65.1%)	81 (61.8%)	Reference		
	AT	61(34.9%)	50 (38.2%)	0.867 (0.542–1.387)	0.355	0.551
Allele	A	241 (68.9%)	200 (76.3%)	Reference		
	T	109 (31.1%)	62 (23.4%)	1.459 (1.014–2.099)	4.162	0.041
rs4786370						
Codominant	CC	115 (65.7%)	72 (55.0%)	Reference	5.227	0.730
	CT	52 (29.7%)	46 (35.1%)	0.708 (0.432–1.160)	1.886	0.170
	TT	8 (4.6%)	13 (9.9%)	0.385 (0.152–0.975)	4.279	0.039
Dominant	CC	115 (65.7%)	72 (55.0%)	Reference		
	CT+TT	60 (34.3%)	59 (45.0%)	0.637 (0.400–1.013)	3.645	0.056
Recessive	CC+CT	167 (95.3%)	118 (89.3%)	Reference		
	TT	8 (4.6%)	13 (10.7%)	0.435 (0.175–1.082)	3.358	0.060
Overdominant	CC+TT	123 (70.3%)	85 (64.9%)	Reference		
	CT	52 (29.7%)	46 (35.1%)	0.781 (0.482–1.267)	1.004	0.316
Allele	C	282 (80.6%)	190 (72.5%)	Reference		
	T	68 (19.4%)	72 (27.5%)	0.636 (0.436–0.930)	5.507	0.019

Abbreviations: CAD, coronary artery disease; CI, confidence interval; OR, odds ratio.

### 3.6 Relationship between plasma IL‐32 levels and *IL*‐*32* SNPs

**FIGURE 4:**
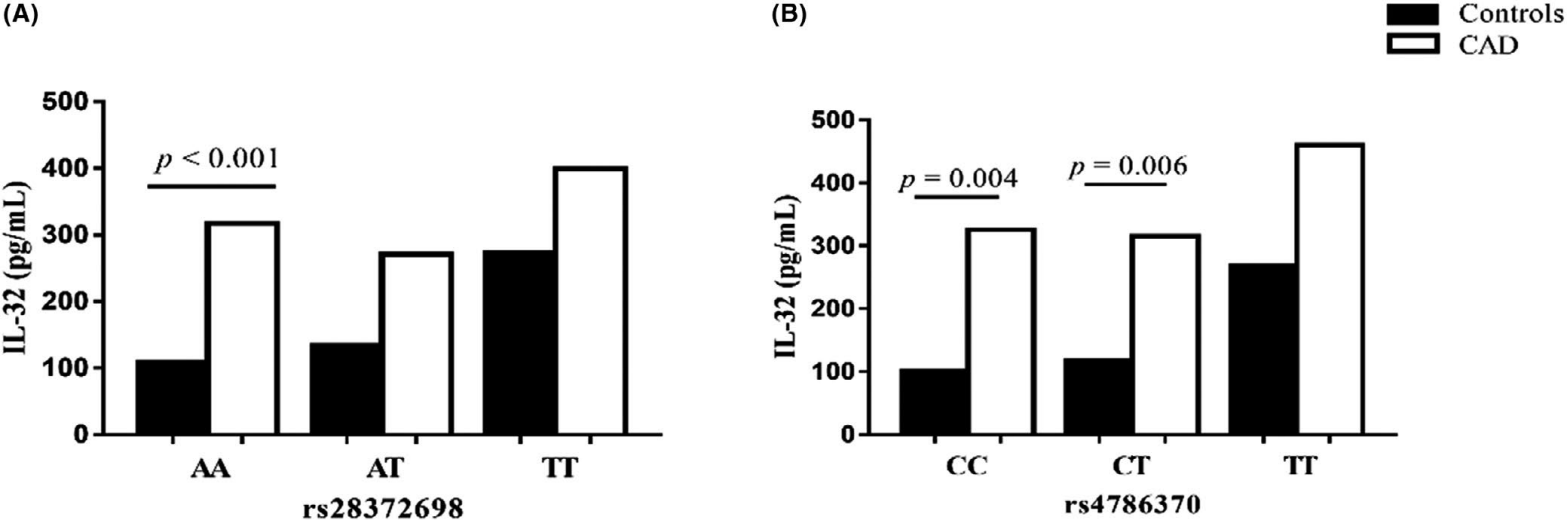
Relationship between plasma IL‐32 levels and *IL*‐*32* SNPs. (A) Association between rs28372698 genotypes and plasma IL‐32 levels in the CAD and control groups. (B) Association between rs4786370 genotypes and the plasma IL‐32 levels in the CAD and control groups. CAD, coronary artery disease

To further explore whether genetic variants could affect plasma IL‐32 levels, we analyzed plasma IL‐32 levels in patients with different genotypes. As presented in Figure 4, no significant association was observed between the different genotypes of SNP rs28372698 and rs4786370 of IL‐32 and plasma IL‐32 levels in the CAD group (*p* = 0.064 for rs28372698, *p* = 0.800 for rs4786370). However, significantly higher levels of plasma IL‐32 were found in the AA homozygotes of rs28372698 in the CAD group than in the control group (*p* < 0.001). Plasma IL‐32 levels of the CC and CT genotypes of rs4786370 in the CAD group were higher than those in the control group (*p* = 0.004 for rs28372698, *p* = 0.006 for rs4786370). We further assessed the effect of *IL*‐*32* SNPs on coronary stenosis severity. The results showed that there was no significant difference between the SNPs and Gensini scores (*p* = 0.561 and *p* = 0.265, respectively).

## 4 DISCUSSION

Our study showed that plasma IL‐32 levels are closely correlated with the degree of coronary artery stenosis and suggests that IL‐32 may be an independent predictor of CAD. To the best of our knowledge, our study is the first to show that the *IL*‐*32* gene (rs28372698 and rs4786370 SNPs) is significantly associated with CAD predisposition in the Chinese Han population.

IL‐32 has been detected in human endothelial cells of atherosclerotic plaques.^20^ As the vascular endothelium is an important mediator in inflammation, IL‐32 as a critical regulator of endothelial cell (EC) functions may promote angiogenesis in endothelial cells,^21^ suggesting that IL‐32 boosts the development of atherosclerosis. IL‐32 is also highly expressed in T cells, which play an important role in the late stages of atherosclerosis characterized by plaque instability and rupture.^22^ A study showed that decrease in IL‐32 levels by siRNA subsequently reduced pro‐coagulant, pro‐inflammatory, and cytokine levels,^23^ suggesting that the association between IL‑32 and cardiovascular risk is even more closely associated with chronic inflammation. Our results revealed significantly higher IL‐32 levels in the diseased than in the control group. Moreover, plasma IL‐32 levels in the control, mild stenosis, and severe stenosis groups were significant different and positively correlated with the Gensini score. The Gensini score has routinely been used as a factor in patients with CAD to evaluate disease severity. This corroborates the findings of Yang et al.,^20^ who demonstrated that a higher plasma IL‐32 level was characteristic of patients with a more severe clinical course of CAD. Furthermore, we investigated the potential of IL‐32 as a biomarker in CAD. Based on ROC analysis, we found that IL‐32 has a superior diagnostic value for CAD and is a better predictor of the progression of coronary arterial lesions.

Genetic factors play a major role in CAD predisposition. Although the relationship between *IL*‐*32* polymorphisms and CAD has not been studied before, their association with several diseases has been previously reported. Previous studies have associated the TT genotype of rs28372698 with an increased risk of systemic lupus erythematosus^8^ and endometrial cancer.^24^ Additionally, rs28372698 has been found to be important in both cancer development and disease pathogenesis.^10^, ^25^, ^26^ Alehagen et al.,^11^ demonstrated that the AA genotype of the *IL*‐*32* SNP rs28372698 was associated with increased risk of both all‐cause and cardiovascular mortality in an elderly community‑living population, which is in contrast to our finding that suggested a strong association of the TT genotype and the T allele of rs28372698 with CAD susceptibility. Another study conducted on a variety of alleles and genotypes showed that *IL*‐*32* SNP rs4786370 may be a risk factor for preeclampsia.^27^ In addition, the C allele of rs4786370 is linked to an increase in HDL‐C levels, which can affect and possibly lower the CVD risk in rheumatoid arthritis patients.^12^ However, this finding is not consistent with our finding that the T allele of rs4786370 reduces CAD risk. Such differences are likely due to differences in ethnicity, sample size, patient selection, clinical heterogeneity, low statistical power, or a combination of these factors.^27^ Furthermore, it is required to clarify the potential relationship between plasma IL‐32 levels and the SNP in question. It has been reported that the TT genotype of rs28372698 leads to increased IL‑32γ expression in thyroid carcinoma.^28^ The TT genotype of rs28372698 has been shown to have significantly reduced IL‐32 levels in colorectal cancer tissue.^29^ Additionally, the CC genotype of rs4786370 has been shown to be associated with slightly increased IL‐32 expression in patients with rheumatoid arthritis.^30^ However, our results showed no significant association between plasma IL‐32 levels and genotypic distribution of rs28372698 and rs4786370 SNPs, which is not consistent with previous reports. We further assessed the effect of the SNPs on the severity of coronary stenosis and found that both SNPs are ineffective in determining CAD severity. Although our study showed that the SNPs were susceptible to CAD, their risk mechanism to CAD remains unknown. Therefore, further studies are warranted to fully evaluate this relationship in different patient populations.

Another interesting finding of our study is the association of plasma IL‐32 levels with PCI. PCI is currently widely used for treating CAD in combination with optimal medical therapy.^31^ It effectively reduces mortality in CAD patients by opening infarct‐related blood vessels and restoring blood flow. Our study showed that IL‐32 levels were significantly higher before PCI than at 7 days post‐PCI. This can be explained by the gradual decline in the inflammatory response due to the restoration of blood flow and statin administration. However, further investigation is needed to validate the effect of statins on IL‐32 levels.

This study is limited in that the size of the group with cardiovascular disease was small which increases the margin of error. Furthermore, the role of *IL*‐*32* SNPs in the progression and prognosis of CAD requires further investigation. Besides, the predictive ability of IL‐32 for the long‐term prognosis and progression of coronary stenosis was not examined because of the cross‐sectional design of our study; therefore, future follow‐up investigations are necessary.

## 5 CONCLUSION

In conclusion, this study is the first to show the association of rs28372698 and rs4786370 with CAD susceptibility in the Chinese Han population. Moreover, it also shows significantly increased plasma IL‐32 levels in patients with CAD and its positive correlation with coronary artery stenosis. We suggest that IL‐32 levels can be a simple and readily available inflammatory marker to predict CAD and assess coronary artery stenosis.

## CONFLICT OF INTEREST

The authors declare that they have no known competing financial interests or personal relationships that could have appeared to influence the work reported in this article.

## AUTHOR CONTRIBUTIONS

SS Jin and XJ Liu searched the literature, selected the study, designed the study, analyzed data, and drafted the article. YY Wang carried out the epidemiological survey, collected the samples, and helped to draft the manuscript. J Yu took part in assessments of quality. MH Jiang interpreted data, and revised the article. All authors read and approved the final manuscript.

## ETHICAL APPROVAL

The study was approved by the ethics committee of the Second Affiliated Hospital and Yuying Children's Hospital of Wenzhou Medical University. Informed consent was obtained from all the individual participants included in the study.

## Data Availability

All data included in this study are available upon request by contact with the corresponding author.
